# KeratoDetect: Keratoconus Detection Algorithm Using Convolutional Neural Networks

**DOI:** 10.1155/2019/8162567

**Published:** 2019-01-23

**Authors:** Alexandru Lavric, Popa Valentin

**Affiliations:** Computers, Electronics and Automation Department, Stefan cel Mare University of Suceava, Suceava 720229, Romania

## Abstract

Keratoconus (KTC) is a noninflammatory disorder characterized by progressive thinning, corneal deformation, and scarring of the cornea. The pathological mechanisms of this condition have been investigated for a long time. In recent years, this disease has come to the attention of many research centers because the number of people diagnosed with keratoconus is on the rise. In this context, solutions that facilitate both the diagnostic and treatment options are quickly needed. The main contribution of this paper is the implementation of an algorithm that is able to determine whether an eye is affected or not by keratoconus. The KeratoDetect algorithm analyzes the corneal topography of the eye using a convolutional neural network (CNN) that is able to extract and learn the features of a keratoconus eye. The results show that the KeratoDetect algorithm ensures a high level of performance, obtaining an accuracy of 99.33% on the data test set. KeratoDetect can assist the ophthalmologist in rapid screening of its patients, thus reducing diagnostic errors and facilitating treatment.

## 1. Introduction

The cornea is the outer layer of the eye, the surface covering the front of the eye. The structural and repair properties of the cornea are essential for its function: protecting the inner contents of the eye, maintaining the shape of the eye, and achieving light refraction.

The cornea is composed of proteins and cells and does not contain blood vessels, unlike most tissues in the human body. The existence of blood vessels may affect its transparency, which in turn may affect the proper light refraction, hence worsening vision. Because there are no blood vessels that supply nutrients in the cornea, tears and aqueous humor (an aqueous liquid) provide nutrients to the cornea.

The cornea is composed of five layers: the epithelium, the Bowman's layer, the stroma, Descemet's membrane, and the endothelium. The first layer, the epithelium, is a cell layer that covers the cornea. This layer absorbs oxygen from tears and passes it to the rest of the cornea. The cornea also contains free nerve endings. Keratoconus is a noninflammatory condition characterized by progressive thinning, deformation, and scarring of the cornea. The pathological mechanisms of keratoconus have been investigated for a long time. Both the genetic and environmental factors have been associated with the disease, but in recent years, a new theory emerges that keratoconus could also have an inflammatory component [1].

However, since many patients with allergies rub their eyes excessively, it has not been clearly established whether eye rubbing is a factor related to the keratoconus pathology but can definitely increase the cornea deformation [2]. Keratoconus eyes are characterized by a cornea that has a progressive cone deformation that gradually reduces the visual accuracy of the patient. Over the years, all kinds of methods have been proposed to correct this condition, such as rigid RGP contact lenses and scleral lenses, or different surgeries to reduce the progression of the disease. In recent years, this disease has come to the attention of many research centers because the number of people diagnosed with keratoconus is continually growing and quick solutions are needed to facilitate both the diagnosis and treatment options.

Hidalgo et al. [3] use a support vector machine (SVM) algorithm that processes 22 parameters in order to differentiate patients suffering from keratoconus from those who are healthy. The algorithm processes corneal topography parameters and measurements of the patient's eye. The development environment used in the study is Weka open source software [4]. The results show an accuracy of 95.2%, which represents a high level of performance. According to the authors, the most suitable algorithm for KTC classification is one of the SVM types. From the obtained results, the reduction of parameters does not affect the performance of the classification algorithm. Contrarily, it may even improve the accuracy in some particular cases, considerably reducing the processing time. The best accuracy was obtained when only 7 parameters were integrated in the model.

Accardo et al. [5] present a keratoconus detection algorithm that uses a neural network (NN). The accuracy achieved by the implemented algorithm is 96.4%. This accuracy was obtained by simultaneously using the topographic parameters of both eyes, which improves the discriminative ability of the neural network.

Valdés-Mas et al. [6] propose the use of an artificial neural network (NN) to predict the evolution of keratoconus. The quality of vision is assessed in the work by analyzing corneal curvature and the astigmatism. The paper proposes a series of models, the best results being obtained through an artificial neural network based on a multilayer perceptron (MLP).

The main benefits of integrating machine learning algorithms in ophthalmology are immense [7]. The machine learning domain is a subbranch of artificial intelligence that, as Arthur Samuel said, “gives computers the ability to learn without being explicitly programmed.”

The first step of a machine learning algorithm is data processing, followed by the training mechanism; then, the algorithm is deployed to predict new features meanwhile monitoring the accuracy. After the algorithm is prepared and trained, the new data (test data) are fed into the network.

Machine learning algorithms have the potential to interrupt classical health screening programs, being able to provide diagnostics in a very short time helping to increase patient care and comfort. One aspect that should not be neglected is the validation of these algorithms, which must be accomplished under objective conditions and by specialized programs.

Fatemeh et al. [8] present an automatic keratoconus detection algorithm by using artificial intelligence. The algorithm uses as input a set of topography images obtained using a Pentacam [9] that has been labelled by specialists in two categories (keratoconus eyes and nonkeratoconus eyes). The disadvantage of the proposed approach is that the training data set contains a small number of images, respectively, 82.

Four groups of classifiers have been tested: multilayer perceptron (MLP) [10], radial basis function network (RBFN) [11], neural network (NN), and support vector machine (SVM) [12]. Simulations show that the results of the classification schemes have roughly the same results in terms of accuracy. The best accuracy obtained was 92.2% for the MLP algorithm, and the lowest accuracy of 84.42% for the SVM algorithm.

In [13], a learning algorithm is presented that is based on a neural network that allows the diagnosis of keratoconus. From the obtained results, the highest accuracy 97.33% was achieved on the test data set. The main disadvantage of the algorithm is the use of a large number of parameters which makes it difficult to implement and test.

Perissutti et al. [14] are among the first to propose the use of a neural network in diagnosing keratoconus. The maximum level of accuracy was 92%. The paper compares the way of detection that integrates both a monocular approach and a binocular approach. The best results are obtained by the authors when the parameters obtained from both eyes of the patient are used in the model.

Arbelaez et al. [15] develop a classification algorithm showing high precision in terms of differentiation between normal eyes and keratoconus-affected eyes. The model also includes posterior corneal surface parameters and corneal thickness. The integration of these parameters significantly improved the sensitivity of the algorithm when considering subclinical keratoconus diagnosis. The classification mechanism may be particularly useful when detecting early signs associated with keratoconus. The accuracy of the classifier developed by the authors was approximately 97.2%. Diagnosing keratoconus at an early stage is very important for the prognosis of the disease.

Maeda et al. [16] combine a discriminatory analysis and a classification tree to analyze data from the corneal topography in order to diagnose keratoconus. The sensitivity of the mechanism was 89%, which is less than the value obtained with an SVM-type algorithm of 95%.

Twa et al. [17] consider modelling the anterior corneal surface with a seventh-order Zernike polynomial and applying a decision tree type to differentiate between a normal eye and a keratoconus one. The sensitivity, specificity, and precision of the method were 92%, 93%, and 94%, respectively.

Chastang et al. [18] integrated a binary decision tree based on the data generated by the corneal topography and achieved a precision of about 95%. The disadvantage of the proposed approach is the existence of a reduced input data set [19]. In conclusion, the algorithms that can be integrated in the keratoconus diagnosis process are as follows: multilayer perceptron (MLP), decision tree (DT), convolutional neural network (CNN), radial basis function network (RBFN), artificial neural network (ANN), and support vector machine (SVM).

The paper is structured as follows: after a short introduction, the motivation that led to this work is presented. In Section 3, the proposed keratoconus detection algorithm (KeratoDetect) is discussed; meanwhile, in Section 4, the performance evaluation of the KeratoDetect algorithm is presented.  Section 5 concludes this work. The main contribution of this work is the integration of a convolutional neural network in the diagnostic process of keratoconus. From the best knowledge of the authors, this approach is the first one. In the scientific literature, a handful of algorithms [3–18, 20] have been presented that use machine learning technologies in keratoconus detection, but none of them obtained such a high level of accuracy.

## 2. Theoretical Background, Symptoms, and Treatment

Keratoconus (KTC) is found in the general population with a ratio of 1 in 2000 persons; also, the incidence among children has increased significantly over the past few years. The number of people diagnosed with keratoconus is rising because more and more people are initially diagnosed when performing the screening for laser refracting surgery which includes an eye topography. Thus, the incidence of keratoconus in the population can be even greater.

The epidemiological reports published in recent years show that in Russia only 0.3 cases per 100,000 people (0.0003%) are reported, in India 2.3% cases, in Israel 2.34%, and 2.5% in Iran [1]. Usually, the illness debuts in the second decade of life and affects both sexes and all ethnicities. Keratoconus usually affects both eyes; however, one eye may be initially affected.

In keratoconus, because of the structural changes caused by the thinning of the cornea, intraocular pressure is no longer provided by the cornea. Thence, the cornea deforms by taking a conical shape. The name of the condition is given by this happening. KTC affects both the young and the elderly, so it is urgently necessary to find new ways of diagnosing so that the keratoconus can be dealt with beforehand.

In advanced cases, most of the time, there is a significant distortion of vision, worsening the quality of life. The most advanced form of vision correction requires a corneal transplant, which involves risks such as rejection and infection. The safest bet is to have a correct diagnosis as soon as possible, so that the patient has the opportunity to undertake treatment that slows the progression of the disease.

A basic proven treatment is the corneal cross-linking that aims to prevent disease progression [2]. The Corneal Cross-Linking (CXL) treatment is aimed at restoring the integrity of the corneal matrix, hence increasing the resistance to keratoconus progression. For the effectiveness of this treatment to be maximum, it must be done when the disease is at an early stage. Thus, it is imperative to correctly diagnose the early stage of keratoconus, which most often has puberty onset.

Continuous development of large sets of ophthalmic data, sustained by improvement of learning algorithms, and the increased processing power have led to heightened interest in applying machine learning algorithms in ophthalmology.

**Figure 1:**
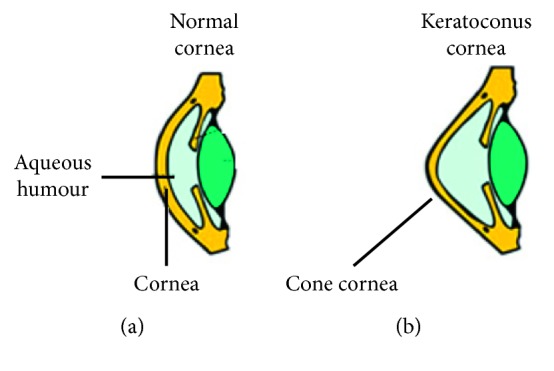
The keratoconus (a) and normal (b) cornea.

The main contribution of this paper is the implementation of an algorithm named “KeratoDetect” that is able to determine if an eye suffers from keratoconus. In Figure 1, the differences between a normal cornea and one affected by keratoconus (KTC) disease (e.g., cone-like cornea) are presented.

## 3. KeratoDetect: Keratoconus Detection Algorithm

The main goal is to implement and test an algorithm that allows keratoconus detection by facilitating the diagnostic process. The algorithm uses a convolutional neural network (CNN). The most used modality to diagnose and confirm keratoconus is to make a corneal topography which is then interpreted by the ophthalmologist specialist. These images will consider the input of KeratoDetect algorithm, within the learning process associated with the convolutional neural network (CNN).

The developed neural network processes the input data (e.g., pixel values of an image representing the corneal topography) using weights on connections between neurons. The learning process involves the continuous adjustment of these weights so as to reduce the error in both the classification and learning processes.

Many learning algorithms use multilayer networks between inputs and outputs. These neural networks allow the identification of features, patterns, and characteristics within the classification relationship. Technological progress has led to the development and refinement of these algorithms, with them being used in many areas of medicine with confidence.

In neural networks, a convolutional neural network (CNN) is one of the main methods of recognizing and classifying images. CNNs are currently used in applications such as object recognition and face detection. A CNN that is capable of diagnosing the keratoconus disease is implemented in this paper.

Classifying images using the CNN algorithm involves an image preprocessing step. The algorithm decomposes the image at the pixel level; the obtained matrix is then applied at the input of the neural network. The algorithm uses color images that have a size of 180 × 240 × 3 pixels. The CNN model assumes that each image passes through a series of kernel convolutional filters, pooling layers, and fully connected (FC) layers; meanwhile, the classification is performed by using the Softmax function to classify an object with probabilistic values between 0 and 1.

**Figure 2:**
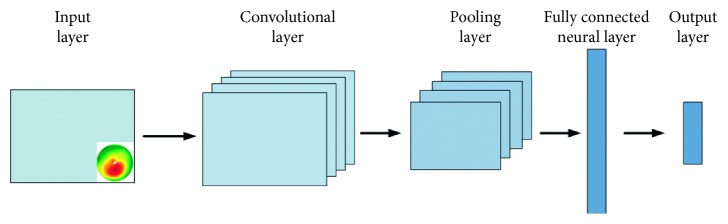
Structure of a CNN.

In Figure 2, the structure of such a CNN is presented. One can observe the input layer, the convention layer, the pooling layer, the fully connected layer, and the output layer.

The convolution layer is the first layer that performs feature extraction from an image applied to the networks' input. Convolution layer keeps the relationship between pixels by learning the characteristics of images using certain filters, thus generating the image matrix.

**Figure 3:**
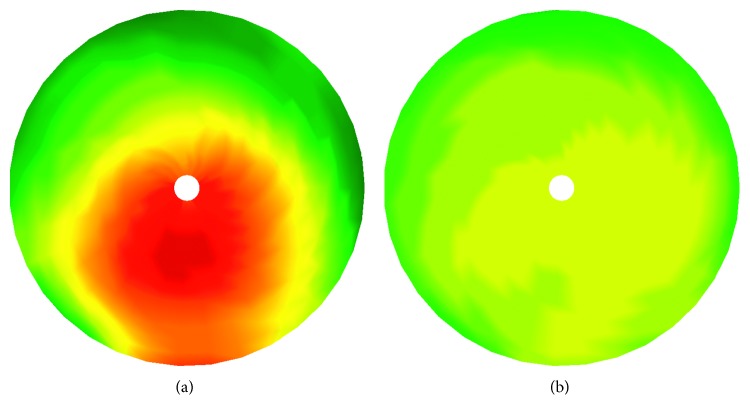
Cornea topographies. (a) Keratoconus eye. (b) Healthy eye.

In Figure 3, two corneal topographies are shown; the former, an eye affected by keratoconus, and the latter, a healthy eye. The colors used in a topography show any changes to the elevation parameter of the cornea. Thus, colors such as red indicate the existence of a high elevation (e.g., the possible existence of a cone). Light colors such as green or yellow show a uniform distribution of the elevation parameter.

Topographic maps use a color scale to identify corneal curvature data. Curved steep areas are displayed in warm colors, such as red and orange, whereas flat bend areas are shown in cold colors, such as green and blue [21]. Topography displays color maps in “absolute” and “normalized” scales. The topography gives an overview of the whole cornea in terms of its curvature. The implemented algorithm processes typical corneal topographies and classifies them into two categories, detecting patterns specific to the keratoconus pathology.

**Figure 4:**
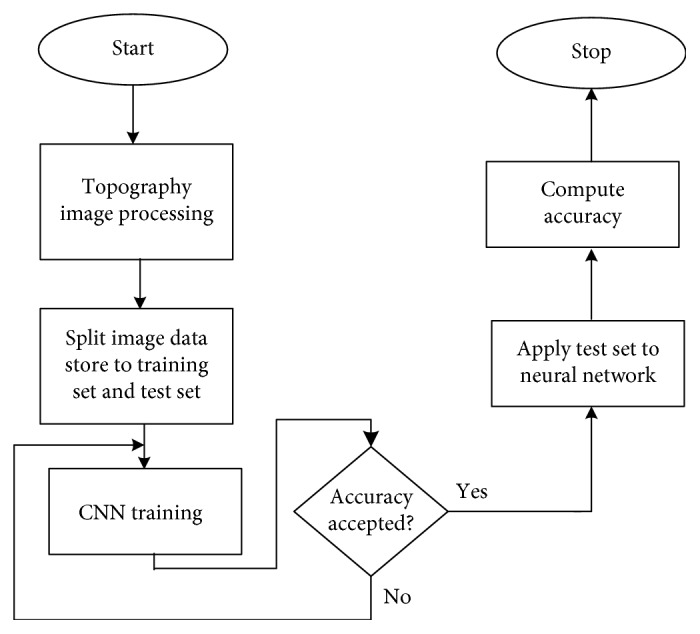
CNN-proposed algorithm.

Figure 4 presents the steps integrated in algorithm proposed by KeratoDetect. The first step involves the preprocessing of the images that will be applied to the input of the algorithm.

Input images must have the same resolution before being applied to the convolutional neural network; hence, they need to be preprocessed. The next step is to divide the images into three sets: one used for neural network training, another data set used for validation, and a third set that will be used to test the implemented algorithm after completing the learning and validation processes.

The next step is to train the neural network using the training image set. Once the accuracy obtained is acceptable, the test image set is applied at the algorithm input. The final parameter analysed and evaluated is the accuracy with which the algorithm was able to correctly classify topography into two categories: keratoconus-affected eye and healthy eye with a normal topography. The accuracy is computed on the test data set.

The colors used in a topography show changes in the elevation parameter of the cornea. Thus, colors nearer to red indicate the existence of a cone. The implemented algorithm processes topical corneal topographies and classifies them into two categories, detecting patterns specific to the keratoconus pathology.

## 4. Performance Evaluation of KeratoDetect

One of the initial problems that we encountered was the hindrance to gathering a large set of corneal topographies that we can apply to the proposed algorithm input. Usually, clinical data are difficult to obtain. So, as a solution, we integrated the SyntEyes KTC model [22]. The model used to generate the input data (e.g., the corneal topographies) is a stochastic one, allowing for the automatic generation of corneal topographies [23].

**Figure 5:**
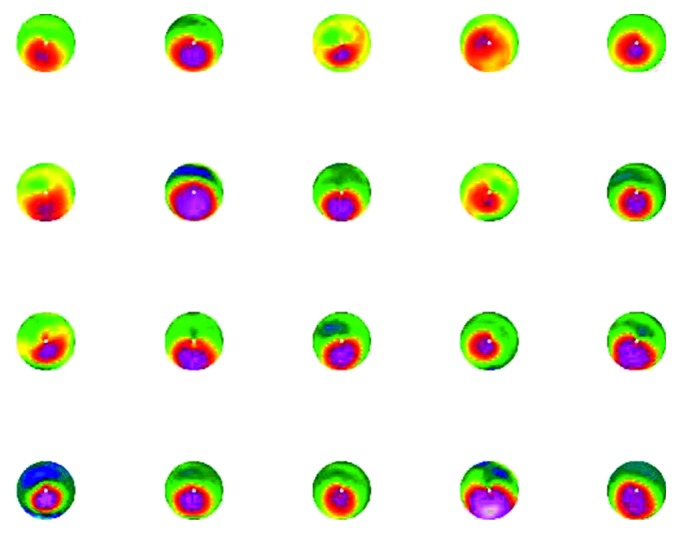
Algorithm input.

In Figure 5, a selection of topographies generated by the SyntEyes is presented.

The SyntEyes model uses as baseline 145 Scheimpflug tomographs, also incorporating eye biometry-related information. Information is processed to reduce the number of integrated parameters followed by a multivariable Gaussian analysis that produces the stochastic model of the keratoconus (KTC) eye. The results of this model are filtered to remove incorrect topographic models either by an automatic or a manual procedure. The evolution and characteristics of keratoconus can be studied and analysed by comparing SyntEyes KTC output with the output provided by the pattern that generates healthy eye with normal topographies because the synthetic data provided by the model resemble current clinical data and can be integrated as learning data within the CNN. The KeratoDetect algorithm was developed using 1500 healthy eye topographies and 1500 topography of the eyes diagnosed with keratoconus. CNN's training set uses 1350 topographies. The algorithm validation data set includes 150 eyes; meanwhile, the test dataset will include 200 eyes on which the performance level will be measured. The CNN algorithm is implemented in Matlab software [24].

**Figure 6:**
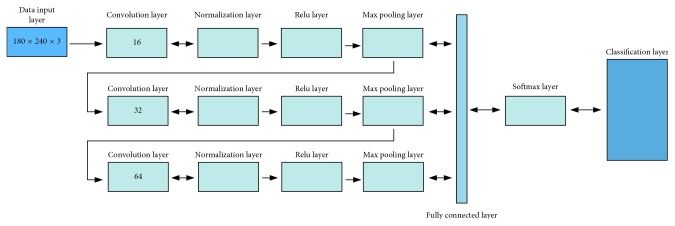
CNN-designed algorithm.

Figure 6 presents the CNN structure implemented in Matlab software. The CNN uses a filter with a size of 3 by 3 pixels for scanning procedure of the training set. The number of neurons used in the convolutional layer is 16 (e.g., the number of filters) neurons that connect to the same region of the input data set.

At the convolutional layer, a padding feature is used in order to create the features map. In our case (a filter of 3), a padding of 1 is used in order to ensure that the spatial output size is the same as the input size. The next integrated layer is a normalization layer that allows the optimization of the network by normalizing the activations.

This layer is followed by a ReLU (rectified linear units) layer. The purpose of this layer is to add nonlinearity after each convolutional layer. This layer lowers the training time of the network and reduces the sensitivity of the CNN.

The next layer of the designed CNN is a max pooling layer which has the role to make a downsampling operation in order to remove redundant information from the layers. This process is used as to increase the number of filters without the supplementary increase in computational power. The max pooling layer returns the maximum values of the defined rectangular regions, using a stride combination with a defined step size of 2 for the training process. The learning rate of the network is set to 0.01.

The next step is to increase the number of filters from 16 to 32 and perform the normalization operations followed by the ReLU layer. After these operations have been fulfilled, the max pooling function is applied. The data are prepossessed again this time by a 64-neuron convolutional layer followed by a normalization and a ReLU layer.

The last layer is a fully connected layer where all the neurons connect to the neurons from the previous layers and exchange data. This layer combines all the features extracted and learned by the previous layers as to identify patterns in the input data. In this layer, all the extracted features are used to classify the corneal topography. The size of the fully connected layer is equal to the classification classes, in our Case 2 (one for keratoconus eyes and one for the normal eyes).

The next layer is a Softmax layer that determines the classification probabilities used by the final classification layer. The classification layer uses an activation function for each input to assign one of the two classes and calculate the loss parameter.

**Figure 7:**
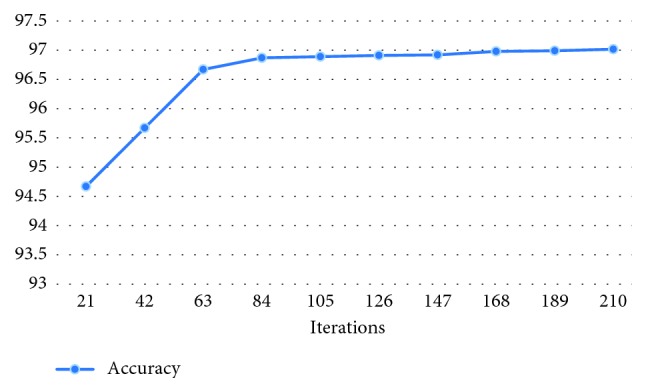
Algorithm accuracy parameter.

Figure 7 shows the accuracy parameter when the number of iterations is varied. One epoch is a set of data used to train the convolutional neural network and coincides with the complete pass of the input data set. One epoch includes 21 iterations. An iteration represents a neural parameter update in the convolutional neural network. The test data include a number of 400 corneal topographies, 200 topographies of keratoconus eyes, and 200 topographies of normal eyes. For a number of 10 epochs (i.e., 210 iterations), we obtain an accuracy on the data test set of approximately 97.01%. The accuracy is computed for the test data set.

**Figure 8:**
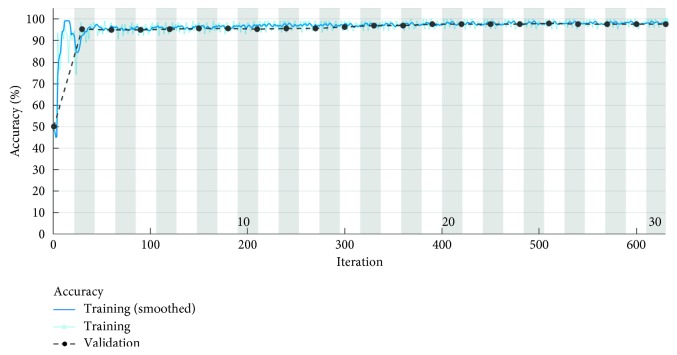
Accuracy parameter for 630 learning iterations.

Figure 8 presents the accuracy parameter when 630 iterations equivalent to 30 epochs are used. Each iteration is an estimate of the gradient, which corresponds to an update of the neural network parameters. In this situation, the developed algorithm has an accuracy of 97.67% for the test data set that includes 400 topographies. By default, the CNN validates the network every 50 iterations by making predictions on the validation data set and calculating the accuracy parameter.

**Figure 9:**
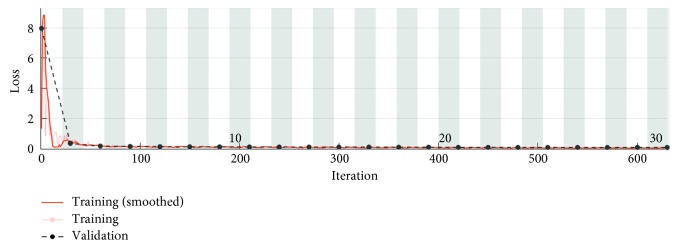
Losses for 630 learning iterations.

Figure 9 shows the loss parameter of the CNN. This parameter represents the cross entropy of the entire CNN. When we increase the accuracy of the network, the loss parameter significantly reduces contributing to the elimination of incorrect topographies classification.

**Figure 10:**
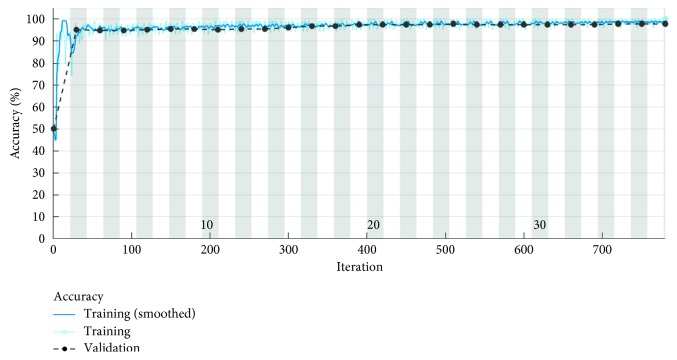
Accuracy parameter for 798 learning iterations.

Figure 10 presents the accuracy parameter for 798 iterations equivalent to 38 epochs.

Each iteration represents an update of the neural network weights, thus increasing the network performance. For the test data set, the developed algorithm has an accuracy of 99.33%. Obviously, in this case, the learning time of the neural network has increased significantly. The algorithm allows detection of keratoconus suspect corneas with very high accuracy.

**Figure 11:**
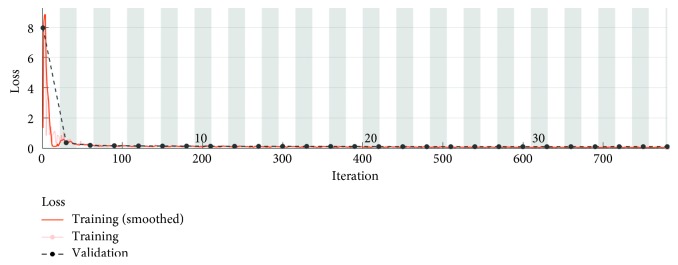
Losses for 798 learning iterations.

In Figure 11 is presented the loss parameter in case the accuracy of the proposed algorithm is 99.33%.

**Figure 12:**
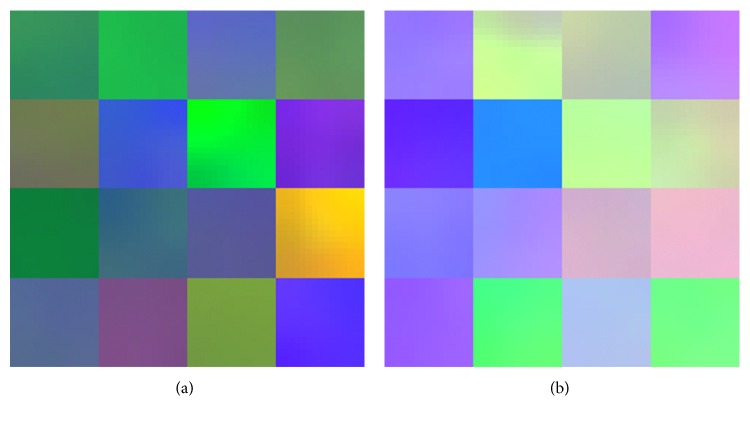
Initial convolution layer weights (before training) (a) and the first convolutional layer weights (after training) (b).

In Figure 12, the initial convolutional layer weights before the learning process are shown as well as the first convolutional layer weights of the CNN after the training process. These weights are used for feature extraction and further processing.

The main advantage of the proposed algorithm is that it ensures the highest level of performance when we compare the obtained accuracy with the accuracy of other algorithms presented in the scientific literature. The obtained level of performance also represents the main contribution of this work.

## 5. Conclusions

The main advantage of the proposed algorithm is that it can be used as an integrated part of the diagnostic process. From the obtained results, we can conclude that the proposed KeratoDetect algorithm ensures a high level of performance. The main contribution of this work is the development and integration in the diagnostic process of an assistant software to help the ophthalmologist. Machine learning algorithms have the potential to interrupt classical medical screening programs, being able to provide diagnostics in a very short time as well as helping to increase patient care and comfort. The contribution of this paper consists in applying a machine learning mechanism to keratoconus disease detection. The high level of performance can come to help the medical staff in correctly diagnosing keratoconus.

Thus, after the ophthalmological consultation, the corneal topography is applied as an input to the already trained neural network, and this will determine whether the patient is suffering from keratoconus or not. By optimizing the parameters associated with the convolutional neural network, the accuracy of the proposed algorithm was increased to 99.33% for the test set. The implemented algorithm processes topical corneal topographies and classifies them into two categories, detecting patterns specific to the keratoconus pathology.

In conclusion, this paper presents the development of a screening tool based on a learning algorithm that automatically detects the keratoconus disease based on corneal topographies.

The algorithm can be implemented in the device that performs the topography as an add-on in order to assist the ophthalmologist in rapid screening of its patients. Although the results show a high level of performance, this mechanism cannot be used as a stand-alone diagnosis procedure but should be seen as an additional tool to help the ophthalmologist who also analyzes other clinical data such as family history, refraction, and corneal shape evolution, and performs lamp examination. In the future, these algorithms will become more efficient and contribute to easy diagnosis of keratoconus and the reduction of corneal transplant cases.

## Data Availability

The data results used to support the findings of this study are presented in this paper.
